# Physical activity and the risk of postmenopausal breast cancer - the Norwegian Women and Cancer Study

**DOI:** 10.1186/1477-5751-13-3

**Published:** 2014-03-01

**Authors:** Kristin Benjaminsen Borch, Eiliv Lund, Tonje Braaten, Elisabete Weiderpass

**Affiliations:** 1 Department of Community Medicine, Faculty of Health Sciences, University of Tromsø, The Arctic University of Norway, Tromsø 9037, N-Norway; 2 Department of Medical Epidemiology and Biostatistics, Karolinska Institutet, Stockholm, Sweden; 3 Department of Research, Cancer Registry of Norway, Oslo, Norway; 4 Folkhälsan Research Centre, Samfundet Folkhälsan, Helsinki, Finland

**Keywords:** Physical activity, Breast cancer, Hormone receptor status, Norway, Women

## Abstract

**Background:**

The relationship between physical activity (PA) throughout life and the risk of postmenopausal breast cancer overall and by estrogen receptor (ER) and progesterone receptor (PR) status, has been reported, but without consistent results. The present study aimed to investigate PA from young age to adulthood in participants of the Norwegian Women and Cancer (NOWAC) Study, in order to determine whether changes in PA level affect the risk of postmenopausal breast cancer.

**Methods:**

1767 invasive breast cancer cases were identified among 80,202 postmenopausal participants of the NOWAC Study during 8.2 years of median follow-up. PA levels at age 14 years, 30 years and at cohort enrollment were obtained via a self-administered questionnaire. Multivariate Cox proportional hazard regression models were used to estimate relative risks and 95% confidence intervals of the risk of postmenopausal breast cancer overall and by ER/PR status.

**Results:**

Risk of postmenopausal breast cancer overall and by ER/PR status was not associated with physical activity level at enrollment. Women with a low PA level at age 30 had an increased risk of ER+/PR + breast tumors (*P for trend* = 0.04) compared to women with a moderate physical activity level at age 30. Women with a low physical activity level at all three periods of life had a 20% significantly reduced risk of postmenopausal breast cancer, as well as a reduced risk of ER+/PR + and ER+/PR- breast tumors, compared with women who maintained a moderate physical activity level. However, when analyses were corrected for multiple tests, the result was no longer statistically significant. The findings were consistent over strata of age, body mass index and use of hormone replacement therapy.

**Conclusions:**

The study results from this large Norwegian cohort do not support an association between physical activity at different periods of life and the risk of postmenopausal breast cancer.

## Background

Breast cancer is the most frequent cancer type among women worldwide in terms of both incidence and mortality [1], and comprises one-quarter of all incident female cancers in Norway [2]. Research has shown evidence of an inverse relationship between physical activity (PA) and the risk of postmenopausal breast cancer [3], with an average observed risk reduction of 25% when comparing the most with the least physically active women in both case–control and cohort studies [4]. However, these findings are not completely consistent; some prospective studies have found either no relationship [5-8], or an increased risk of postmenopausal breast cancer with increasing amount of PA [9-11]. Despite the heterogeneity between studies on the topic, in their joint Second Expert Report published in 2007 [12], and in updates from 2008 [13] and 2010 [14], the World Cancer Research Fund and the American Institute for Cancer Research concluded that PA probably reduces the risk of postmenopausal breast cancer. A recent meta-analysis of 31 prospective studies concluded that there is a significant, reduced risk of overall breast cancer of 12%. It further concluded that this reduction is consistent across categories of menopausal status, body mass index (BMI), hormone receptor status of tumor, tumor stage, type and intensity of PA, and the periods of life in which PA was reported [15]. However, there is some evidence that the impact of lifestyle factors like PA on breast cancer risk may vary by tumor characteristics, such as estrogen receptor (ER) and progesterone receptor (PR) status [4]. Moreover, studies on the association between PA at different periods of life and the risk of postmenopausal breast cancer have shown inconsistent results [3]. Only a few studies included data on PA from young age to early adulthood, i.e. from age 14 to 24, and this data mainly reflected recreational or leisure PA [8,16-20]. None of these studies found an inverse relationship, except one case–control study which found a weak inverse relationship between PA and risk of postmenopausal breast cancer [20]. Assessment of PA from young age to adulthood can give important insight into how PA early in life affects the risk of postmenopausal breast cancer.

In 2005, Margolis et al. analyzed the relationship between risk of premenopausal breast cancer and PA at different periods of life in the Women’s Lifestyle and Health Study. The Women’s Lifestyle and Health Study is a prospective study of women in Norway and Sweden [9], and includes the cohort of the Norwegian Women and Cancer (NOWAC) Study. To complement this work, the aim of the present report was to investigate PA from young age to adulthood in participants of the NOWAC study, to determine whether changes in PA level affect the risk of postmenopausal breast cancer overall and by ER/PR status.

## Results

A total of 80,202 postmenopausal women from the NOWAC study were followed for an average of 8.2 years, constituting 648,731 person-years. Mean age at enrollment for the study sample was 48 years. During follow-up, 1,767 women were diagnosed with breast cancer (mean age at diagnosis: 59.7 years). Information on ER status was available for 79% of breast cancer cases; of these 83% were ER + and 17% were ER-. PR status was available for 79% of breast cancer cases, of which 63% were PR + and 37% were PR-. Seventy-four percent of the women reported moderate, high or very high PA levels at enrollment. This proportion was even higher for the same PA levels at age 14 years (85.7%) and age 30 years (86.0%) (Table 1). Among women who reported being inactive at age 14, 5.3% were still inactive at age 30, 77.1% who reported being active at age 14, were still active at age 30, and 8.7% went from active at age 14 to inactive at age 30. Of the women who reported being active at age 14, 64.3% were still active at enrollment, 21.4% went from active to inactive and 4.7% were inactive at age 14 and at enrollment (Table 1).

**Table 1:** Physical activity (PA) level and changes in PA level across the three periods of life among 80,202 postmenopausal women in the Norwegian Women and Cancer Study

		**PA level (%)**				
	**N**	**Very low**	**Low**	**Moderate**	**High**	**Very high**
At age 14^a^	77 623	2.2	12.1	34.6	34.1	17.0
At age 30^a^	78 477	1.7	12.2	42.9	32.0	11.1
At enrollment	80 202	5.2	20.9	41.8	25.6	6.4
		**Changes in PA level**^**b **^**(%)**				
		**Inactive-no change**	**Active to inactive**	**Inactive to active**	**Active-no change**	
Age 14 to age 30	77 272	5.3	8.7	9.0	77.1	
Age 14 to enrollment	77 623	4.7	21.4	9.6	64.3	
Age 30 to enrollment	78 477	8.3	17.8	5.6	68.3	

^a^Missing at age 14, 3.2%; age 30, 2.2%, compared to enrollment.

^b^Inactive: very low or low PA level. Active: Moderate, high or very high PA level.

BMI at enrollment was inversely associated with being active at enrollment. Physically active women were over-represented among never and former smokers, and physically active women who were ever smokers tended to smoke less cigarettes per day, had lower alcohol consumption, used less hormone replacement therapy and reported less cardiovascular disease and diabetes compared to inactive women. Physically active and inactive women had similar ages at menarche, first birth and menopause, and a similar history of oral contraceptive use (Table 2).

**Table 2:** Characteristics of the study sample at enrollment by physical activity (PA) level among 80,202 postmenopausal women from the Norwegian Women and Cancer Study

	**PA level**				
	**Very low**	**Low**	**Moderate**	**High**	**Very high**
N	4196	16 773	33 538	20 564	5131
Age (years)	49.1	48.8	48.3	48.1	47.5
Duration of education (years)	11.3	12.2	12.3	12.4	11.6
Weight (kg)	70.8	68.8	66.1	64.5	63.1
Height (cm)	165.7	166.2	166.2	166.4	166.0
Body mass index (kg/m^2^)	25.8	24.9	23.9	23.3	22.9
Smoking status:					
Never smoker	29.5	34.8	36.2	35.9	33.1
Former smoker	29.6	31.0	32.6	34.6	32.5
Current smoker	41.0	34.2	31.3	29.5	34.2
Pack-years (in former and current smokers)	8.5	6.5	5.5	5.2	5.6
Alcohol consumption (g/day)	3.5	3.5	3.4	3.5	3.1
Age at menarche	13.2	13.3	13.3	13.3	13.4
Oral contraceptive use (ever)	46.2	45.0	45.2	44.7	46.5
Age at first birth (years)	23.4	23.9	23.9	23.9	23.5
Nulliparous	10.8	9.0	8.1	8.0	7.6
Age at menopause (years)	47.6	48.3	48.4	48.3	48.1
Hormone replacement therapy use (current)	27.9	29.0	27.6	28.1	24.5
Self-reported cardiovascular disease (yes)	9.1	5.4	3.9	3.8	4.4
Self-reported diabetes (yes)	3.0	1.7	1.3	1.1	1.2

### Risk of postmenopausal breast cancer and PA level at age 14, age 30 and at enrollment

In adjusted multivariable models, there was no association between PA level at enrollment or at age 30 and risk of postmenopausal breast cancer (Table 3). In contrast, compared to women with a moderate PA level at age 14, women with a low PA level at age 14 had a decreased risk of postmenopausal breast cancer (relative risk, RR 0.80; 95% confidence interval, CI: 0.68, 0.95; *P for trend* = 0.008). The same comparison using women with a very low PA level at age 14 rendered an RR of 0.77 (95% CI: 0.54, 1.10).

**Table 3:** Incidence of postmenopausal breast cancer and physical activity (PA) level at age 14, age 30 and at enrollment among 80,202 postmenopausal women from the Norwegian Women and Cancer Study

	**PA level**					
	**Very low**	**Low**	**Moderate**	**High**	**Very high**	***P ***_**trend**_
**At age 14**						
N cases^b^	32	170	609	599	302	
RR (95% CI)^a^	0.77 (0.54, 1.10)	0.80 (0.68, 0.95)	1.0 (ref)	1.02 (0.91, 1.14)	1.04 (0.90, 1.19)	0.008
**At age 30**						
N cases^b^	34	206	746	548	189	
RR (95% CI)^a^	1.16 (0.82, 1.64)	.99 (0.85, 1.16)	1.0 (ref)	0.97 (0.87, 1.09)	0.91 (0.78, 1.08)	0.23
**At enrollment**						
N cases	105	401	722	443	96	
RR (95% CI)^a^	1.06 (0.86, 1.30)	1.05 (0.93, 1.19)	1.0 (ref)	1.04 (0.92, 1.17)	0.91 (0.73, 1.12)	0.40

^a^Multivariable model with RR and 95% CI used the attained age as the underlying time variable. Covariates adjusted for in the model were height, body mass index, smoking status, smoking duration (pack years), age at menarche, use of oral contraceptives, age at first birth, parity, use of hormone replacement therapy, self-reported disease and history of breast cancer in the participant’s mother.

^b^The total number of breast cancer does not add up due to by missing information on PA level at younger ages, age 14 to age 30 n = 77,272, age 14 to enrollment n = 77,623 and age 30 to enrollment n = 78,477.

RR: relative risks; CI: confidence intervals.

### ER/PR status and PA level at age 14, age 30 and at enrollment

No statistically significant associations were observed between PA level at enrollment and ER/PR status of breast tumors (Table 4). At age 30 a significant trend (*P for trend =* 0.04) of increased risk of ER+/PR + breast tumors was observed among women with a very low PA level compared to those with a moderate PA level (Table 4). However, compared to women with a moderate PA level at age 14, those with a low PA level at age 14 showed a trend of reduced risk for ER+/PR + (*P for trend =* 0.02) and ER+/PR- (*P for trend =* 0.02) breast tumors. Analysis of women with unknown ER/PR status showed no significant associations with PA level (data not shown). We found no indication that increasing PA beyond the moderate PA level was inversely associated with ER/PR status.

**Table 4:** Estrogen receptor (ER) and progesterone receptor (PR) status of breast tumor and PA level at age 14, age 30 and at enrollment among 1,767 breast cancer cases in the Norwegian Women and Cancer Study

	**PA Level**						
**ER/PR status**	**N cases**	**Very low**	**Low**	**Moderate**	**High**	**Very high**	***P ***_**trend**_
**ER+/PR+**^**a**^	872	56	188	373	208	47	
PA at enrollment		1.07 (0.81, 1.42)	0.94 (0.79, 1.12)	1.0 ref	0.95 (0.80, 1.12)	0.87 0(.65, 1.19)	0.49
PA at age 14		0.68 (0.40, 1.16)	0.74 (0.58, 0.95)	1.0 ref	1.02 (0.87, 1.20)	1.03 (0.85, 1.25)	0.02
PA at age 30		1.53 (1.00, 2.36)	1.10 (0.89, 1.40)	1.0 ref	0.95 (0.81, 1.12)	0.90 (0.71, 1.13)	0.04
**ER+/PR-**^**a**^	294	17	73	100	84	20	
PA at enrollment		1.32 (0.78, 2.22)	1.43 (1.06, 1.94)	1.0 ref	1.40 (1.04, 1.87)	1.32 (0.81, 2.13)	0.98
PA at age 14		0.83 (0.36, 1.88)	0.62 (0.40, 0.98)	1.0 ref	0.92 (0.70, 1.22)	1.28 (0.93, 1.76)	0.02
PA at age 30		1.21 (0.53, 2.75)	0.84 (0.57, 1.26)	1.0 ref	1.00 (0.76, 1.30)	1.03 (0.70, 1.51)	0.68
**ER-/PR-**^**a**^	206	8	47	91	52	8	
PA at enrollment		0.70 (0.34, 1.46)	1.03 (0.72, 1.47)	1.0 ref	0.94 (0.67, 1.32)	0.57 (0.28, 1.18)	0.51
PA at age 14		1.60 (0.73, 3.49)	1.06 (0.67, 1.70)	1.0 ref	1.10 (0.78, 1.54)	1.15 (0.76, 1.74)	0.94
PA at age 30		0.56 (0.14, 2.28)	0.78 (0.48, 1.27)	1.0 ref	0.90 (0.66, 1.24)	0.81 (0.50, 1.30)	0.97

^a^Multivariable model with relative risks and 95% confidence intervals used the attained age as the underlying time variable. Covariates adjusted for in the model were height, body mass index, smoking status, smoking duration (pack years), age at menarche, use of oral contraceptives, age at first birth, parity, use of hormone replacement therapy, self-reported disease and history of breast cancer in the participant’s mother.

### Overall risk of postmenopausal breast cancer and changes in PA level

A significant increased risk of postmenopausal breast cancer (RR 1.18; 95% CI: 1.00, 1.39) was found among women who changed their PA level from active to inactive between age 14 and age 30. Women who changed their PA level in the opposite direction; i.e. from inactive to active, had a significant reduced risk of postmenopausal breast cancer (RR 0.81; 95% CI: 0.67, 0.97). In contrast, women who were inactive at age 14 and age 30 had a significantly reduced risk of postmenopausal breast cancer (RR 0.78; 95% CI: 0.62, 0.99). Women who were inactive from age 14 to enrollment had a non-significant reduced risk (RR 0.82; 95% CI: 0.64, 1.03), compared to women who were active at all three periods of life (Table 5). We did not detect any significant association between changes in PA level from age 30 to enrollment and risk of postmenopausal breast cancer, even after stratification by age (data not shown).

**Table 5:** Postmenopausal breast cancer risk overall and by estrogen receptor (ER) and progesterone receptor (PR) status of breast tumors and changes in physical activity (PA) level among 1,767 breast cancer cases in the Norwegian Women and Cancer Study

	**N cases**	**Overall Breast cancer**	**N cases**^**a**^	**ER+/ PR+**	**N cases**^**a**^	**ER+/ PR-**	**N cases**^**a**^	**ER-/PR-**
**Changes in PA level**^**b**^				**RR (95% CI)**^**c**^		**RR (95% CI)**^**c**^		**RR (95% CI)**^**c**^
**Age 14 to age 30**								
Inactive	75	0.78 (0.62, 0.99)	37	0.79 (0.56, 1.10)	9	0.53 (0.27, 1.03)	10	0.91 (0.48, 1.73)
Active to inactive	163	1.18 (1.0, 1.39)	96	1.43 (1.15, 1.78)	27	1.10 (0.74, 1.65)	11	0.72 (0.39, 1.32)
Inactive to active	125	0.81 (0.67,0 .97)	56	0.74 (0.56, 0.97)	20	0.73 (0.46, 1.15)	21	1.16 (0.73. 1.82)
Active	1334	1.0 ref	650	1.0 ref	229	1.0 ref	155	1.0 ref
**Age 14 to enrollment**								
Inactive	72	0.82 (0.64, 1.03)	34	0.75 (0.53, 1.06)	9	0.63 (0.32, 1.24)	10	1.06 (0.56, 2.03)
Active to inactive	419	1.06 (0.95, 1.20)	203	1.00 (0.85, 1.18)	78	1.29 (0.98, 1.69)	44	1.07 (0.75, 1.52)
Inactive to active	130	0.79 (.66, 0.95)	59	0.71 (0.54, 0.93)	20	0.71 (0.45, 1.13)	21	1.12 (0.70, 1.77)
Active	1091	1.0 ref	550	1.0 ref	179	1.0 ref	124	1.0 ref
**Age 30 to enrollment**								
Inactive	146	1.06 (0.89, 1.27)	79	1.15 (0.91, 1.46)	24	1.05 (0.69, 1.62)	11	0.72 (0.39, 1.33)
Active to inactive	347	1.04 (0.92, 1.18)	160	0.95 (0.79, 1.13)	64	1.27 (0.95, 1.69)	43	1.18 (0.83, 1.67)
Inactive to active	94	1.01 (0.82, 1.25)	55	1.19 (0.90, 1.57)	12	0.77 (0.43, 1.37)	11	1.01 (0.54, 1.87)
Active	1136	1.0 ref	562	1.0 ref	188	1.0 ref	136	1.0 ref

^a^The total number of breast cancer does not add up caused by missing information on PA at younger ages, age 14 to age 30 n = 77,272, age 14 to enrollment n = 77,623 and age 30 to enrollment n = 78,477.

^b^Inactive: very low or low PA level, Active: moderate, high or very high PA level.

^c^The multivariable model with RR and 95% CI used the attained age as the underlying time variable. Covariates adjusted for in the model were height, body mass index, smoking status, duration of smoking (pack years), age at smoking initiation, age at menarche, use of oral contraceptives, age at first birth, parity, use of hormone replacement therapy, self-reported disease and history of breast cancer in the participant’s mother.

RR: relative risks; CI: confidence intervals.

### ER/PR status and changes in PA level

The analyses of ER/PR status and changes in PA level at the three periods of life revealed a significant increased risk of 43% for ER+/PR + breast tumors among women who were active throughout all three periods of life compared to women who went from being active to being inactive between age 14 and age 30 (Table 5). The results also suggested that changing from inactive to active between age 14 and age 30 (RR 0.74; 95% CI: 0.56, 0.97) and between age 14 and enrollment (RR 0.71; 95% CI: 0.54, 0.93) protected against ER+/PR + breast tumors. We found no evidence that changes in PA level had any effect on ER+/PR- or ER-/PR- breast tumors (Table 5), nor on breast tumors with unknown hormone receptor status (data not shown).

Results from crude models were not significantly different than those from multivariate models (data not shown). All multivariate models were adjusted for BMI, but results were almost identical to those from models without adjustment for BMI. Homogeneity tests confirmed that there were no statistical differences between the magnitudes of the effect estimates. We assessed the potential effect modification of BMI and hormone replacement therapy use by conducting stratified analyses. These analyses revealed no statistically significant interaction of BMI or hormone replacement therapy use on the relationship between PA level at enrollment or changes in PA level and risk of postmenopausal breast cancer overall, or by ER/PR status (data not shown). The analyses stratified by different periods of enrollment did not change the estimates (data not shown). Finally, the Holms procedure for multiple testing showed that no estimates reached statistical significance, neither for different periods of life, or for different ER/PR status of breast cancer tumors (data not shown).

## Discussion

In this Norwegian cohort study, there was no consistent, significant relationship between PA level and postmenopausal breast cancer overall, or by ER/PR status. We found an increased risk of postmenopausal breast cancer overall, and of ER+/PR + breast tumors among women who went from active to inactive between age 14 and age 30. Furthermore, among women who changed from inactive to active at the same ages, the opposite was true; they had a decreased risk of postmenopausal breast cancer overall, and of ER+/PR + breast tumors compared to women who remained physically active throughout all three periods of life. Furthermore, we observed that women with a very low PA level at age 30 had an increased risk of ER+/PR + breast tumors compared to women with a moderate PA level at the same age.

We also observed a statistically significant 20% reduction in the risk of postmenopausal breast cancer among women with PA levels that were low at age 14, and remained low throughout adulthood, as compared to women who were either moderately active at age 14, or had a moderately active lifestyle throughout adulthood (i.e. at age 30 and at enrollment, which occurred between the ages of 34 and 70 years). This unexpected association was confirmed in the analysis of ER/PR status, which revealed a decreased risk of ER+/PR + and ER+/PR- breast tumors, though this was not the case for any of the other combinations of ER/PR status. Taken together these findings are inconsistent, and the risk of false-positive findings only strengthens the uncertainty. Analysis for multiple testing confirmed that none of the estimates reached statistical significance, indicating that the inverse relationship between PA level and postmenopausal breast cancer was not strong in our study, and this must be considered when reviewing the results.

The strengths of our study include the prospective cohort study design, long follow-up period and the completeness of follow-up data, which minimizes the potential for selection bias. The ascertainment of postmenopausal breast cancer and of the ER/PR status of breast tumors was performed through the linkage to the national cancer registry, which is virtually complete. Our study sample was taken from a source population that is considered representative of Norwegian general female population 34 to 70 years of age [21]. The availability of PA level over different periods of life and the possibility to investigate changes in PA level is an important strength of this study. The most important risk factors for postmenopausal breast cancer were taken into account as confounding factors in our analyses, including age at menarche, age at first birth, parity, age at menopause, history of breast cancer in the participant’s mother, exogenous hormone use, BMI, height, alcohol consumption [22,23] and cigarette smoking [24-26]. Furthermore, a sensitivity analysis was carried out in which we adjusted for BMI at age 18 years, which minimized potential residual confounding of unmeasured, or other unknown factors in the relationship under study.

Our study also has some limitations. The lack of a clear risk pattern may be partly due to the fact that PA level was self-reported, and collected all at once for three different periods of life. Therefore this information was based on the participant’s capability to recall PA levels correctly; incorrect recall could have led to misclassification that might have influenced the true exposure status early in life. Moreover, the changes in PA level at age 14, age 30 and at enrollment may not be entirely representative of the 15–20 years in between. Indeed, elderly women have been shown to overestimate PA levels earlier in life [27,28]. This would result in non-differential misclassification, and could therefore bias the results toward the null. Our measures to capture the effect of PA on a specific disease may be considered crude, since there was no information on type, duration, frequency or intensity of PA. However, the PA level in the present study covers the total amount of PA, and is meant to reflect a comprehensive measure. Finally, the low number of women with very low PA levels at age 14 led to a low number of breast cancer cases in this group, and reduced the statistical power of the analysis. This also represents a limitation of the study and should be considered when evaluating the results.

ER and PR status was missing for 21% of the women in the present analysis. Therefore analyses on ER/PR status of breast tumors had less power than analyses on postmenopausal breast cancer overall. Our choice of age 53 as a cut-off for age at menopause ended up excluding women that were likely menopausal. Also, we used PA levels reported at enrollment for all three time periods for women who became postmenopausal during follow-up (and thus were included only from that point in time). Finally, lack of controlling for unknown factors, and the fact that information on potential confounding factors was only collected at enrollment, may have introduced residual confounding.

The original aim of the NOWAC study was to investigate the association between breast cancer and oral contraceptive use, with no specific emphasis on PA other than as a possible confounding factor. Although the correlation between our PA measure and the reference method [29] was modest, this same scale of PA level was inversely associated with all-cause, cardiovascular and cancer mortality in the NOWAC cohort, indicating a face validity of sensitivity to detect major disease trends and variations [30]. The internal validity of self-reported information on use of oral contraceptives, parity, duration of education [31] and use of hormone replacement therapy [32], has been investigated in the NOWAC Study, and is considered valid.

Our results did not corroborate findings from other studies that suggested PA protects against postmenopausal breast cancer [4,7,18,33-38]. In a recent review of 73 studies (both case–control and prospective cohort studies), only 40% found a statistically significant risk reduction, which was 25% on average when comparing women with high and low PA levels [4]. Furthermore, the strongest inverse associations were found with recreational PA, PA throughout life, PA after menopause, and PA of moderate and vigorous intensity performed regularly [4,39]. A recent meta-analysis of 31 prospective studies found an inverse relationship between PA and breast cancer, with 12% risk reduction and a dose–response relationship. This association was stronger for women with a BMI less than 25 kg/m^2^, premenopausal women, and for ER-/PR- breast tumors [15]. However, our results on postmenopausal breast cancer are in agreement with previous investigations on postmenopausal breast cancer or hormone receptor status and total PA by self-administered questionnaire, which detected no association, or only weak associations after adjusting for confounding factors [5,6,40-45]. In a previous prospective study of Norwegian women, the findings in postmenopausal women did not confirm any effect of PA during leisure time or at work [46]. Others have found a modest inverse effect of total PA level [47]. Conversely, another investigation has found a non-significant trend of increased risk of postmenopausal breast cancer with increasing PA [10].

In principle, our findings indicate the same trend, suggesting that low PA level at age 14 has a modest protective effect on the risk of postmenopausal breast cancer overall, and on ER+/PR + breast tumors. These results are in contrast with those of The Women’s Health Initiative, which investigated PA at age 18 years and risk of postmenopausal breast cancer and found no association either overall or by hormone receptor status. However, they found an inverse effect of PA at age 35 [18]. A case–control study on PA early in life found that strenuous PA at age 14–22 was associated with 45% reduction in the risk of postmenopausal breast cancer [16], whereas a prospective study failed to detect any effect of sports at young ages [8]. A review examining PA in adolescence and young adulthood found an almost 20% risk reduction with moderate/vigorous recreational PA, despite variation in the populations and the inclusion of both case–control and prospective cohort designs [48].There are conflicting findings regarding the most relevant time period PA should take place to reduce the risk of postmenopausal breast cancer. One study found limited support for an inverse association between PA during adolescence and throughout life and the risk of postmenopausal breast cancer [17]. Others have found that recreational PA during the reproductive and postmenopausal years is the most relevant period for reducing the risk of postmenopausal breast cancer [49], as well as sustained PA throughout life [4,17,50]. Studies using a single point of PA measure did not find any association [40,43,44], which strengthens the argument that baseline PA is not sufficient to predict the risk of postmenopausal breast cancer over longer periods. The long latency between carcinogenic exposures and the manifestation of breast cancer [51,52] supports the argument that PA patterns need to be assessed over a woman’s lifetime.

The inverse relationship of PA on hormone receptor-positive breast tumors has been reported [7,38,53,54]. We observed a weak inverse relationship between PA and an increased risk of ER+/PR + breast tumor status, when very low and low PA levels were compared with the moderate PA level at age 30. This association is in line with findings from the majority of investigations published to-date [3,38,47,55,56], and strengthens the hypothesis that PA may act through hormonal pathways, by reducing the cumulative exposure to ovarian hormones [39,57]. Studies have reported an inverse relationship for the combination of ER+/PR+, ER+/PR-, ER-/PR- status and high PA, but not for moderate PA levels [7], whereas others have found stronger associations for ER- breast tumors [36,58], and an inverse relationship of PA with both ER+/- and PR+/- breast tumors [18,54,59,60]. PA is likely to act through multiple pathways, which could explain the inconsistency in previous findings, together with a limited power to analyze ER and PR status in breast tumors and PA [38,53,54,61], and the heterogeneity assessing PA which makes it difficult to compare results between studies.

Some studies have confirmed that the association between PA and breast cancer is not modified by BMI [5,19,34,35,37,47,62-64], but not all of them [18,19,40,65]. Several studies reported no modification effect of use of hormone replacement therapy [18,19,37,47,65], though a few did find an effect [36,45]. Although BMI and use of hormone replacement therapy may be related to breast cancer, the aforementioned results suggest that PA is independently associated with breast cancer.

## Conclusion

Our study did not detect a consistent inverse effect of PA on the risk of postmenopausal breast cancer in this large cohort of Norwegian women. For future research, information on PA during follow-up is essential. Despite the results of the present study, PA remains an important modifiable lifestyle factor with the potential to reduce the risk of postmenopausal breast cancer.

## Methods

The NOWAC Study has been previously described in detail [21,31]. In short, at cohort enrollment (1991, 1996–1997 and 2003–2004), participating women completed an extensive questionnaire, including questions on PA, dietary habits, smoking status and habits, alcohol consumption, education, reproductive history, height and weight, exogenous hormone use, and previous illnesses. The Regional Ethical Committee and the Norwegian Data Inspectorate approved the NOWAC Study. All women gave written informed consent prior to their participation in the study.

### Study sample

A total of 122,857 women who participated in the NOWAC Study were initially eligible for the present analysis. We excluded all women with prevalent cancer at enrollment (n = 4620), those who died within the first year of follow-up (n = 265) and those with missing information on PA level at enrollment (n = 12,313). Women who reported their age at menopause at the time of enrollment or during follow-up (available from second questionnaire for some of the women) were categorized as postmenopausal. All other women, including those with missing information on age at menopause, were categorized as postmenopausal once they reached 53 years of age during follow-up, i.e., 53 years of age was used as a proxy for age at menopause. This cut-off was based on the definition used in the Million Women Study, and later in the NOWAC study [66-68]. Based on these criteria, we excluded all premenopausal women (n = 12,235). We further excluded women with missing information on any relevant covariates. This left a final study sample of 80,202 postmenopausal women; of these 3.2% had missing information on PA levels at age 14, and 2.2% at age 30.

### Self-reported PA level

PA level at age 14, age 30 and at enrollment (ages 34–70) was assessed by self-report on an ordinal scale of 1 to 10. PA was defined in the questionnaire as follows: *“By physical activity we mean activity both at work and outside work, at home, as well as training/exercise and other physical activity, such as walking, etc. Please mark the number that best describes your level of physical activity; 1 being very low and 10 being very high”.* The PA scale used for this study has recently been validated for the assessment at enrollment [29], and refers to the total amount of PA across different domains, including frequency, duration and intensity in one global score. Moderate, but significant (*P* <0.001) Spearman’s rank correlation coefficients were found (range: 0.36-0.46) between the PA level at enrollment and concurrent outcomes from criterion measures of a combined sensor monitoring heart rate and movement. The scale ranged from 1 (very low) to 10 (very high), and corresponded to mean values of 0.8 and 3.4 hours/day of moderate/vigorous PA, respectively, with a linear increase (*P for trend* <0.001), and appeared valid to rank PA level in a Norwegian population of women [29]. The PA levels at age 14 and 30 could not be validated in a concurrent design. The PA levels used in the present analysis were collapsed as follows: very low (levels 1 and 2), low (levels 3 and 4), moderate (levels 5 and 6), high (levels 7 and 8) and very high (levels 9 and 10), which were created to resemble those used in earlier analyses [9,69]. We further categorized women based on changes in PA level over time, from age 14 to age 30 and further to enrollment (age 34–70 years). We then dichotomized the PA levels very low or low as inactive, and PA levels of moderate, high and very high as active for each period of life considered. We compared PA levels between age 14 and age 30, age 14 and enrollment, and between age 30 and enrollment. This led to four categories of changes in PA level: those who remained inactive, those who went from active to inactive, those who went from inactive to active, and those who remained active.

### Covariates

Information collected at enrollment included age, duration of education, weight, height, BMI, smoking history (including smoking status: never, former, current; duration and quantity of cigarettes smoked/day (pack-years) and age at smoking initiation, alcohol consumption (grams/day), age at menarche, use of oral contraceptives, age at first birth, parity, age at menopause and use of hormone replacement therapy. Self-reported illnesses included cardiovascular diseases (history of heart failure, myocardial infarction, angina pectoris and hypertension) and diabetes mellitus. Information on history of breast cancer in the participant’s mother (yes/no) was also collected. Information on prevalent cancer was obtained through linkage to the Cancer Registry of Norway.

### Follow-up

Person-years were calculated from start of follow-up for women who were postmenopausal at study enrollment, and from age at menopause (either reported or 53 years) for all other women, until date of diagnosis, emigration, death, or end of the study period (December 31st 2009), whichever occurred first. We obtained information on date of death or emigration from the Norwegian National Population Register, and on cancer diagnosis and ER and PR status of breast tumors through linkage to the Cancer Registry of Norway. Cancer diagnoses were coded according to the 10th revision of the International Statistical Classification of Diseases, Injuries and Causes of Death. The main endpoint in this study was incidence of invasive breast cancer (C50), and ER and PR status of breast tumors was classified as follows: ER+/PR+, ER+/PR-, ER-/PR+, ER-/PR- and unknown.

### Statistical analysis

Characteristics of the study participants were examined by PA level and breast cancer incidence. Cox proportional hazard regression models were used to estimate the hazard ratio as a measure of relative risk (RR) with corresponding 95% confidence intervals (CI). In specific analyses of ER and PR status, breast tumors without a specific ER or PR status were considered censored observations. There were too few ER-/PR + breast tumors to allow for a meaningful analysis, therefore no corresponding results are presented. Tests for trend were estimated using the collapsed five-level scale and entered as a continuous term in the Cox proportional hazards regression models.

The proportional hazard assumption was checked using Schoenfeld residuals and Kaplan-Meier log (-log) survival plots, which suggested no evidence of deviation from proportionality. In the multivariable Cox regression models, confounders adjusted for in the different models were height (cm), BMI (kg/m^2^), smoking status (never, former, current), smoking habits, which included smoking duration (years), quantity (pack-years as the total number of years a smoker smoked 20 cigarettes/day) and age at smoking initiation combined as one variable (age at smoking initiation ≥20 years and current smoker; age at smoking initiation ≥20 years and former smoker; age at smoking initiation <20 years, 0–19 pack-years and current smoker; age at smoking initiation <20 years and former smoker; and finally ≥20 pack-years, age at smoking initiation <20 years and current smoker), alcohol consumption (none, 0.1-3.9, 4.0-10.0 and >10.0 grams/day), age at menarche (years), use of oral contraceptives (ever/never), age at first birth (<20, 20–25, >25 years), parity (nulliparous, 1, 2, ≥3 children), use of hormone replacement therapy (current/never), self-reported cardiovascular disease (yes/no) and diabetes mellitus (yes/no), and history of breast cancer in the participant’s mother (yes/no).

Duration of education and total energy intake were not included in the final models, as they were not appreciably related to breast cancer after adjustment for the other confounders in the NOWAC cohort [70] or the present analysis. In each comparison, the group with moderate PA level was set as the reference group. In the analysis of changes in PA level, the group of women who remained active over all three periods of life was set as the reference group. Analyses were done for a crude model (including age), and for multivariate models with and without BMI as a covariate. Additionally the models were stratified by BMI (<25 and ≥25 kg/m^2^), use of hormone replacement therapy (ever/never) and age (30–39, 40–49, 50–59, and ≥60 years) for change in PA between age 30 and enrollment. Use of hormone replacement therapy may modify the association between BMI and risk of postmenopausal breast cancer [71,72], and further influence the effect estimation of PA level on the risk of postmenopausal breast cancer in the models that included BMI. Therefore this was examined before the analyses were undertaken. No significant mean differences were found between BMI in never users compared to ever users of hormone replacement therapy, thus further stratification was not performed.

Homogeneity of the effect estimates in the different models was evaluated using the Wald test to compare different regression estimates. Information regarding BMI at 18 years of age was available for 95% of the participants, thus we carried out a sensitivity analysis; BMI at age 18 has no effect on out risk estimates, therefore this variable was not included in the main models. Finally, we conducted Holm’s multiple-test procedure to protect against false-positive conclusions when analyzing subgroups of breast cancer. Analyses were conducted using STATA version 12.0, special edition (StataCorp, College Station, Texas, USA), with all statistical tests two-sided and conducted at the 0.05 significance level.

## Competing interests

The authors declare that they have no competing interests.

## Authors’ contributions

KBB carried out and interpreted the statistical analysis and drafted the manuscript. TB contributed to the statistical analysis and interpretation of the data and critical revision of the manuscript. EL is the principle investigator and designed the NOWAC Study and contributed with critical revision of the manuscript. EW contributed with the design of the present study and critical revision of the manuscript. All authors read and approved the final manuscript.
